# Carbapenem-resistant bacteria in an intensive care unit during the coronavirus disease 2019 (COVID-19) pandemic: A multicenter before-and-after cross-sectional study

**DOI:** 10.1017/ice.2021.144

**Published:** 2021-04-16

**Authors:** Renato Pascale, Linda Bussini, Paolo Gaibani, Federica Bovo, Giacomo Fornaro, Donatella Lombardo, Simone Ambretti, Giulia Pensalfine, Lucia Appolloni, Michele Bartoletti, Sara Tedeschi, Fabio Tumietto, Russell Lewis, Pierluigi Viale, Maddalena Giannella

**Affiliations:** 1 Infectious Diseases Unit, Department of Medical and Surgical Sciences, IRCCS Azienda Ospedaliero-Universitaria di Bologna, Bologna, Italy; 2 Operative unit of Microbiology, IRCCS Azienda Ospedaliero-Universitaria di Bologna, Bologna, Italy; 3 Hospital Pharmacy, IRCCS Azienda Ospedaliero-Universitaria di Bologna, Bologna, Italy

## Abstract

**Objective:**

To assess the incidence of colonization and infection with carbapenemase-producing Enterobacteriaceae (CPE) and carbapenem-resistant *Acinetobacter baumannii* (CR-Ab) in the ICUs of our city hospitals before and during the coronavirus disease 2019 (COVID-19) pandemic.

**Methods:**

We conducted a multicenter, before-and-after, cross-sectional study to compare the rates of colonization and infection with CPE and/or CR-Ab in 2 study periods, period 1 (January–April 2019) and period 2 (January–April 2020). Incidence rate ratios (IRRs) and 95% confidence intervals (CIs) of weekly colonization and infection rates for each period were compared for the 2 study periods using Poisson regression. Weekly trends in the incidence of colonization or infection for each study period were summarized using local weighted (Loess) regression.

**Results:**

We detected no significant change in either IRR and weekly trend in CPE colonization and infection during the 2 study periods. A shift from KPC to other CPE mechanisms (OXA-48 and VIM) was observed during period 2. Compared to period 1, during period 2 the IRR of colonization and infection with CR-Ab increased 7.5- and 5.5-fold, respectively. Genome sequencing showed that all CR-Ab strains belonged to the CC92/IC2 clonal lineage. Clinical strains clustered closely into a single monophyletic group in 1 of the 3 centers, whereas they segregated in 2 different clusters in the other 2 centers, which strongly indicates horizontal transmission.

**Conclusions:**

Our findings indicate the need to conduct infection control activities targeted against the spread of antimicrobial resistance between and within hospitals during the COVID-19 pandemic, and if necessary, remodulating them according to the new organizational structures imposed by the pandemic.

Before the emergence of the coronavirus disease 2019 (COVID-19) pandemic, the world was already demanding immediate, coordinated, and ambitious actions to avert the antimicrobial resistance (AMR) crisis and its related economic and health consequences. In particular, carbapenem-resistant bacteria are major causes of infection morbidity and mortality in several European countries, and Italy is one of the main areas affected.^1^

During the first pandemic wave, some authors have observed decreased antibiotic susceptibility in local pathogens compared to pre–COVID-19 periods.^2^ Others have reported an increased risk of carbapenem-resistant infections in patients hospitalized with COVID-19 for both intensive care unit (ICU) and non-ICU settings.^3–4^ However, a systematic assessment of colonization and infection with these strains before and during COVID-19 pandemic has not been reported yet.

We performed a before-and-after cross-sectional study to compare the incidence of colonization and infection with carbapenem-resistant bacteria in the ICUs of our city hospitals before and during the COVID-19 pandemic.

## Methods

### Study design

We performed a multicenter, before-and-after, cross-sectional study to compare the incidence of colonization and infection with carbapenemase-producing Enterobacteriaceae (CPE) and/or carbapenem-resistant *Acinetobacter baumannii* (CR-Ab) during 2 study periods: (1) January–April 2019 and (2) January–April 2020. Data sources were the hospital administrative records and the databases of the microbiology laboratories. The study was approved by our ethics committee (Comitato Etico Indipendente di Area Vasta Emilia Centro, no. 283/2020/Oss/AOUBo).

### Setting

The study was conducted in 2 teaching hospitals (Policlinico di Sant’Orsola and Bellaria Hospital) and 1 tertiary-care, nonteaching hospital (Maggiore Hospital). Policlinico di Sant’Orsola (PSO) is a 1,420-bed university hospital with an average of 72,000 admissions per year. It includes 2 main adult ICUs: 1 mixed medical and postsurgical unit (PSO-ICU1) with 22 beds and 1 unit dedicated to patients undergoing major heart–thorax–vascular surgery (PSO-ICU2) with 21 beds. Maggiore Hospital (MH) is the second main city hospital, with 870 beds. The hospital comprises 2 adult ICUs: MH-ICU3 specialized in posttraumatic resuscitation with 11 beds and MH-ICU4 attending patients with both medical and surgical conditions with 9 beds. Bellaria Hospital (BH) is a teaching hospital with 320 beds. It hosts the regional reference center for neurology and neurosurgery with 1 ICU (BH-ICU5) with 12 beds.

During the COVID-19 pandemic, changes in usual activities, number of beds and staff occurred in all of these ICUs; these are described in Supplementary Table 1 (online). A unique infection control program, with the same leadership and scope, was active in all of the ICUs during the 2 study periods. Among other activities, universal screenings for CPE by rectal swabs at ICU admission, once weekly thereafter, and at ICU discharge were performed as previously described.^5^ In mechanically ventilated patients, surveillance for CPE and CR-Ab (tracheal aspirates or bronchoalveolar lavage) was also performed once weekly on respiratory samples. The screening practices did not change between the 2 study periods.

### Population

All consecutive adult patients (aged ≥18 years) admitted to ICU were included in the study. Patient days were computed from the date of ICU admission to that of ICU discharge, and patients were included all the times they were admitted to an ICU during the study periods.

### Variables and definitions

The endpoint variables were colonization and/or infection with CPE or CR-Ab. Colonization and/or infection was assessed from the date of ICU admission to ICU discharge. It was considered only once at the time of the positive sample of the first incident. Carbapenem resistance was defined according to European Committee on Antimicrobial Susceptibility Testing (EUCAST) criteria.^6^ Colonization was defined as bacterial isolation without clinical signs or symptoms suggestive of infection. Infection was defined according to Centers for Disease Control and Prevention (CDC) criteria.^7^

Other variables of interest included age, sex, type of ICU, ICU admission, and ICU discharge dates, total numbers of rectal swabs and respiratory specimens collected during the ICU stay, and antibiotic consumption according to defined daily dose (DDD) per 100 patients of meropenem and ceftazidime-avibactam. We collected data on days of mechanical ventilation (MV) in a subset of patients admitted to the ICUs of PSO from February 1 to April 30, 2019, for the prepandemic period (period 1), and from February 1 to April 30, 2020, for the pandemic period (period 2).

### Microbiology

During the study period, CPE strains were collected in accordance to the active surveillance screening and following regional guidelines.^8^ Briefly, rectal swabs were collected from hospitalized patients and screened for CPE by combining culture-based detection and identification of carbapenemase type, as previously described.^9^

Whole-genome sequencing of CR-Ab isolates collected from blood cultures and respiratory samples was performed as previously described.^10^ Briefly, libraries were prepared by the DNA Nextera XT sample preparation kit and were sequenced using the Illumina ISeq100 platform (Illumina, San Diego, CA) with a 2×150 paired-end run. All reads were evaluated using FastQC (Barbraham Bioinformatics) and then assembled with SPAdes software (University of California–San Diego) with careful settings. Antimicrobial resistance, sequence type (ST), and plasmid content were evaluated using a CGE server (https://cge.cbs.dtu.dk/services/MLST/). The clonal relationships among the CR-Ab isolates were investigated using core-genome single-nucleotide polymorphism (SNP) analysis as previously described.^11^

### Statistical analysis

Data for continuous variables are presented as mean (standard deviation, SD) or median (interquartile range, IQR), and categorical variables are presented as total number and proportion. A 2-sided *P* value of <.05 was considered statistically significant. Weekly crude rates of CR-Ab and CPE colonization or infection per number of total ICU patients’ days with 95% confidence intervals (CIs) were calculated for the 2 corresponding study periods. Numerators were derived from the cohort of positive colonization or infection cases and denominators were derived from the cumulative patient admission days in the ICU for each study period. Incidence rate ratios (IRRs) and 95% CIs of weekly colonization and infection rates for each period were compared for the 2 study periods using Poisson regression. All analyses were performed with the Incidence2 tool kit developed as part of RECON (https://www.repidemicsconsortium.org) using R version 4.0.2 software (R Foundation for Statistical Computing, Vienna, Austria; 2020).

## Results

The number of beds, the characteristics of ICUs, and the number of personnel that changed during the 2 study periods are presented in Supplementary Table 1 (online). The number of beds increased from 75 to 131, with most beds (N = 96, 73.3%) dedicated to patients with COVID-19 in period 2.

During period 1, 1,252 patients were admitted to an ICU 1 or more times. Of these, 766 (61.2%) were male, and the median age was 65 years (IQR, 49–75). The overall number of ICU admissions was 1,345; the median length of ICU stay was 2 days (IQR, 1–5); and the overall number of patient days was 7,817. During period 2, 1,151 patients were admitted to an ICU 1 or more times: 724 (62.9%) were men, and the median age was 65 years (IQR, 54–74). Overall, there were 1,367 ICU admissions, the median length of ICU stay was 3 days (IQR, 1–8), and there were 8,700 patient days. In the subset of assessed patients for the length of mechanical ventilation (MV), the overall days of MV were 711 in period 1 and 1,976 in period 2. The median days of MV were 1 day (IQR, 1–4) in period 1 and 12 days (IQR, 7–21.5) in period 2 (*P* < .001) (Table 1).

**Table 1.** Comparison of Characteristics of ICU Stay During the 2 Study Periods

Characteristic	Period 1	Period 2	*P* Value
No. of patients admitted to ICU	1,252	1,151	
Sex, male, no. (%)	766 (61.2%)	724 (62.9%)	
Age, median y (IQR)	65 (49-75)	65 (54-74)	
Overall no. of ICU admissions	1,345	1,367	
ICU stay, d (IQR)	2 (1-5)	3 (1–8)	<.005
Overall patient days	7,817	8,700	
Overall days of MV	711	1,976	
Days of MV, median (IQR)	1 (1–4)	12 (7–21.5)	<.001

**Note.** ICU, intensive care unit; IQR, interquartile range; MV, mechanical ventilation.

**Fig. 1.**
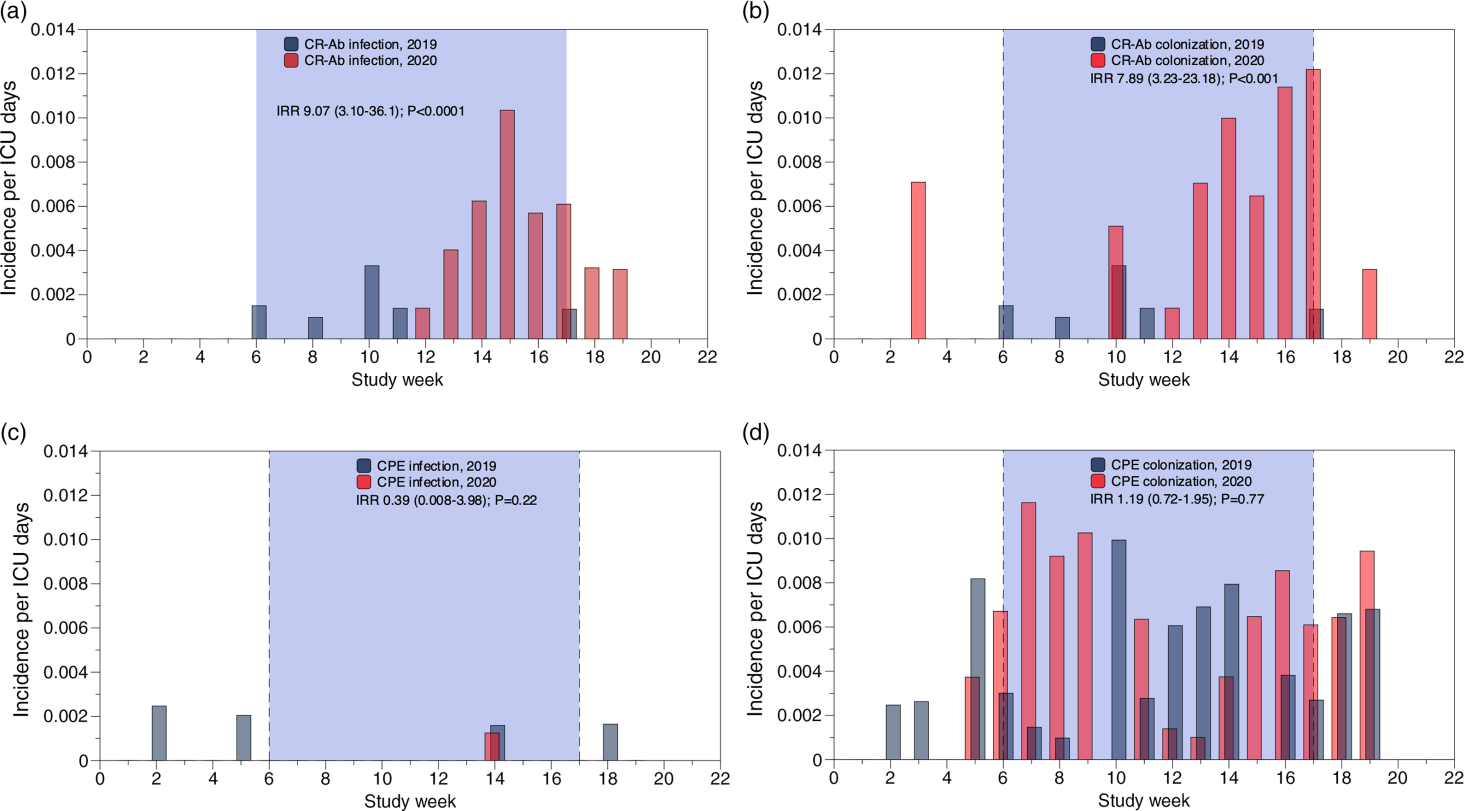
Weekly incidence of CR-Ab infection (a) and colonization (b); CPE-infection (c) and colonization (d). The incidence rate ration (IRR) was calculated only using data from week 6 through week 17 (blue shading between vertical dotted lines) corresponding to the activation of COVID units in 2020.

For CPE, the overall incidences of colonization were 47.3 per 10,000 patient days in period 1 and 40.2 per 10,000 patient days in period 2, and the overall incidences of infection were 3.83 per 10,000 patient days in period 1 and 2.29 per 10,000 patient days in period 2 (Fig. 1, panel c and d). For the subset of patients assessed for days of MV (187 in period 1 and 125 in period 2) the CPE incidences of colonization were 4.2 per 10,000 patient days in period 1 and 5.6 per 10,000 patient days in period 2. We detected no significant change in either the incidence rate ratio (IRR) or the weekly trend in CPE colonization between the 2 study periods (Fig. 1, panel d).

For CR-Ab, the overall incidences of colonization were 5.1 per 10,000 patient days in period 1 and 36.8 per 10,000 patient days in period 2, while the overall incidences of infection were 5.1 per 10,000 patient days in period 1 and 26.4 per 10,000 patient days in period 2. For the subset of patients assessed for days of MV in period 2, the CR-Ab overall incidence of colonization per 10,000 patient days was 11.2 and the CR-Ab overall incidence of infection was 7.2. At the same time, in period 1 no infection or colonization by CR-Ab was reported. Compared to period 1, during period 2 the IRRs of colonization and infection with CR-Ab increased 7.5 and 5.5-fold, respectively (Fig. 1, panel b and a). These increases were observed from week 10 to week 20.

A comparison of colonization and infection episodes with CPE and CR-Ab between the 2 study periods is shown in Table 2. For CPE, although there was no difference in colonization and infection rates between the 2 study periods, during period 2, we observed a decrease in the prevalence of KPC in favor of OXA-48– and VIM–producing strains. CR-Ab colonization accounted for 5 cases (0.4%) in period 1 and 32 cases (2.3%) in period 2. Among them, infection developed in 4 cases (0.3%) in period 1 and 23 cases (1.7%) in period 2.

**Table 2.** Comparison of Colonization and Infection Episodes During the 2 Study Periods

Characteristic	ICU Admissions Period 1 (N=1345), No. (%)	ICU Admissions Period 2 (N=1367), No. (%)	*P* Value
**No. of samples processed**			
Rectal swabs	750	1,118	
Respiratory specimens (BAL and or BAS)	2,632	2,469	
**Carbapenemase-producing Enterobactericeae**			
Colonization	37 (2.8)	35 (2.6)	.81
Rectal	36/37 (97.3)	32/35 (91.4)	
Respiratory	1/37 (2.7)	3/35 (8.5)	
**Mechanism of resistance**			.006
KPC	33/37 (89.2)	22/35 (62.8)	
OXA-48	0	6/35 (17.1)	
VIM	2/37 (5.4)	7/35 (20)	
NDM	2/37 (5.4)	0	
Infection	3 (0.2)	2 (0.1)	.68
BSI	2/3 (70)	1/2 (50)	
LRTI	1/3 (30)	1/2 (50)	
**Carbapenem-resistant** ***A. baumannii***			
Colonization	5 (0.4)	32 (2.3)	<.001
Rectal	0	5/32 (15)	
Respiratory	5/5 (100%)	16/32 (50)	
Other	0	16/32 (34)	
Infection	4 (0.3)	23 (1.7)	<.001
BSI	0	9/23 (39)	
LRTI	4/4 (100%)	14/23 (60.8)	

Note. BAL, bronchoalveolar lavage; BAS, bronchoalveolar aspirate; KPC *Klebsiella pneumoniae* carbapenemase–producing; NDM, New Delhi metallo-β-lactamase–producing; BSI, bloodstream infection; LRTI, lower respiratory tract infection.

Overall, 21 CR-Ab strains isolated from respiratory and blood samples during period 2 were assessed for clonal relationship. A summary of the phenotypic and genotypic characteristics of these strains is shown in Supplementary Table 2 (online). Clinical data of patients from which the strains were isolated are provided in Supplementary Table 3 (online). Antimicrobial susceptibility profiles showed that all clinical CR-Ab isolates were resistant to carbapenems (imipenem and meropenem), fluoroquinolone and aminoglycosides. Moreover, 13 isolates (61.9%) were resistant to trimethoprim–sulfamethoxazole, whereas all CR-Ab isolates were susceptible to colistin. Genetic analysis showed that all strains carried bla_*OXA-23*_ carbapenemase and 14 (66.6%) of 21 CR-Ab strains harbored bla_*TEM1D*_. In addition, aminoglycoside resistance determinants armA and aadA1 were found in all isolates, whereas sulfonamide resistance genes *sul1* and *sul2* were found in 13 isolates.

The MLST analysis showed that all CR-Ab strains belonged to the CC92/IC2 clonal lineage. In detail, the predominance of CR-Ab isolates collected from hospital 1 (PSO) belonged to the ST195 (*N* = 15, 93.8%), while all isolates collected from hospital 2 and 3 were assigned to the ST369 following the Oxford MLST scheme.

A phylogenetic tree based on the core genome SNPs analysis of CR-Ab genomes demonstrated that all ST195 clinical strains isolated from hospital 1 (PSO) clustered closely into a single monophyletic group, whereas CR-Ab strains belonged to the ST369 segregated in 2 different clusters (Fig. 3).

As for antibiotic consumption, there was an increase in the use of meropenem with 88.59 and 108.39 DDD per 100 patients in period 1 and period 2, respectively. Even for ceftazidime/avibactam we observed an increase in consumption in period 2 over period 1: 9.77 versus 7.39 DDD per 100 patients, respectively.

## Discussion

We found no difference in the IRRs of colonization and infection with CPE during the pre-COVID-19 period and the COVID-19 period, whereas the IRR of CR-Ab increased significantly during the COVID-19 period. When was assessed clonal relationship, we found 3 different clusters, 1 for each of the 3 hospitals involved.

During the first wave of the COVID-19 pandemic, several factors could have favored the emergence and spread of antimicrobial resistance in hospitals. First, the overload of patients hospitalized with suspected or proven COVID-19 who then required intensive care assistance may have favored patient-to-patient transmission.^12^ Second, the overuse of antibiotics for suspected bacterial coinfections or superinfections may also have contributed to the emergence and spread of antimicrobial resistance.^13^ Finally, the possible delay in providing culture and sensitivities results from the microbiology laboratories due to the COVID-19 overload may have contributed to inappropriate antibiotic treatment.^14,15^

Although some authors have reported an increase in antimicrobial resistance during COVID-19 period compared with prior periods,^2^ we found no increase in the IRR of colonization or infection with CPE, which was endemic in the hospitals of our city. However, there was a change in the mechanisms of resistance with a decrease in the prevalence of KPC in favour to OXA-48– and VIM–producing strains. This shift was probably not related to the pandemic. The introduction of ceftazidime-avibactam in the therapeutic armamentarium since the beginning of 2019 could partially explain this finding. Indeed, there was a concomitant increase in the consumption of such antibiotics during period 2. However, the design and objective of our study did not allow us to explore this hypothesis further.

The IRRs of colonization and infection with CR-Ab, which were sporadically observed in our hospitals before pandemic, increased significantly. Notably, 9 of the 21 strains assessed for clonal relationship were isolated from patients transferred from other hospitals. Of these, 7 were from a city heavily affected by COVID-19 pandemic in our region (Piacenza, Italy).

Experts have recently emphasized the need to follow antimicrobial stewardship principles during the COVID-19 pandemic.^16^ Our study also highlights the importance of enhancing infection control activities directed against antimicrobial resistance. Indeed, although we observed an increase in the use of meropenem in period 2, which is a key factor in the selection of carbapenem-resistant bacteria,^17^ the clonal relationship clustered per hospital strongly indicates the occurrence of horizontal transmission once the strain was introduced into the hospital.

Our study has several limitations. First, we did not collect patient-level data; thus, we were unable to investigate the impact of clinical variables such as underlying conditions, clinical severity, and therapeutic management on the risk of carbapenem-resistant infection. In addition, administrative data may have been inaccurate in determining the occurrence of hospital-acquired infections. However, we reduced this limitation by using highly accurate laboratory data and revising the clinical charts and hospital electronic records of patients with positive cultures for CPE and/or CR-Ab. Second, period 2 included weeks that did not officially overlap the COVID-19 pandemic, which could have diluted the number of patient days and hence the overall incidence of CPE and CR-Ab colonization and/or infection episodes. However, the analysis per week clearly reveals excess CR-Ab infections. Finally, the local epidemiology and the need to reorganize the capacity, spaces and staff of our ICUs during pandemic may limit the generalizability of our results.

**Fig 2.**
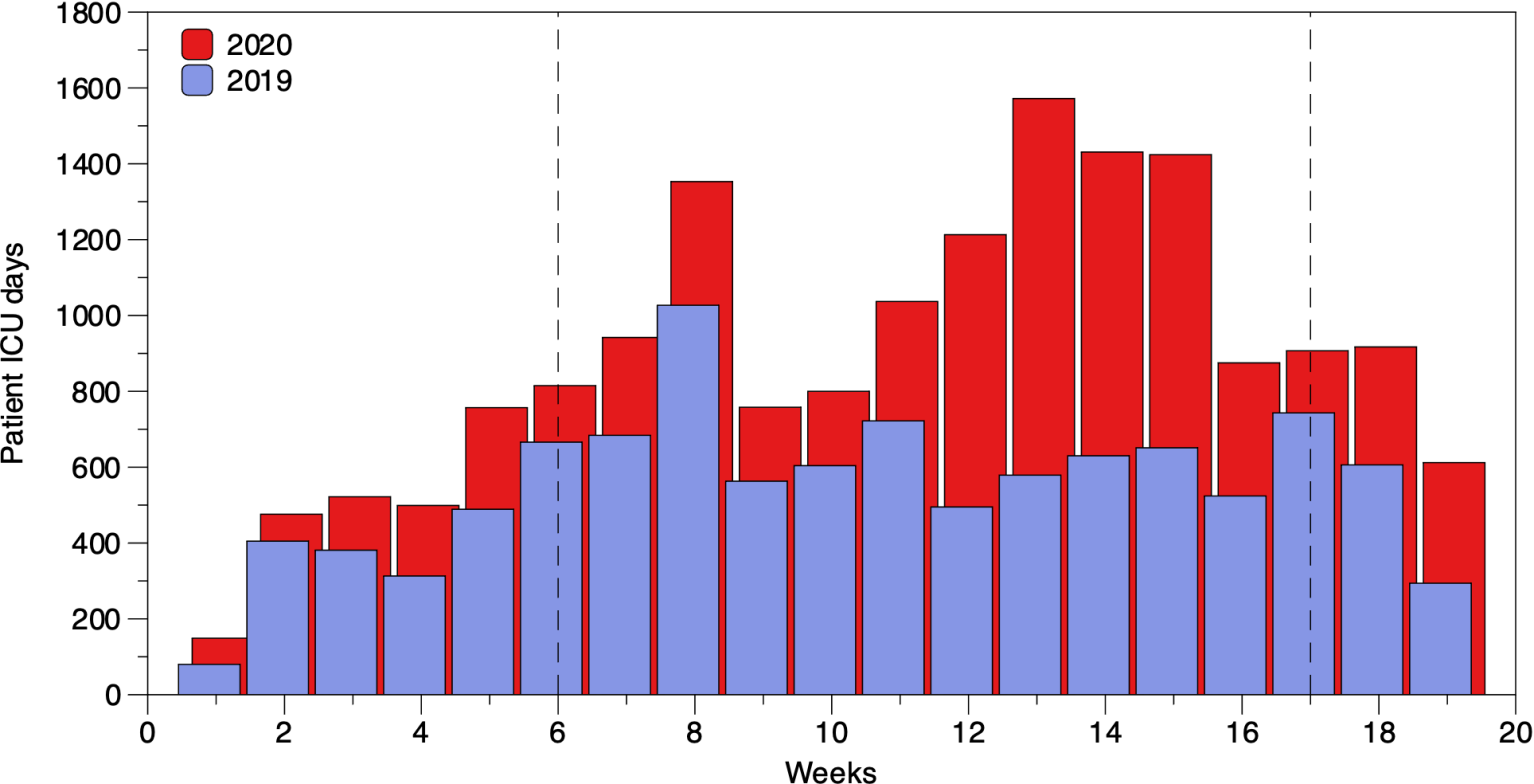
Trends in ICU patient days from January through April in 2019 and 2020. Vertical dotted lines indicate the period with COVID units used to calculate the incidence rate ratio of infection.

**Fig. 3.**
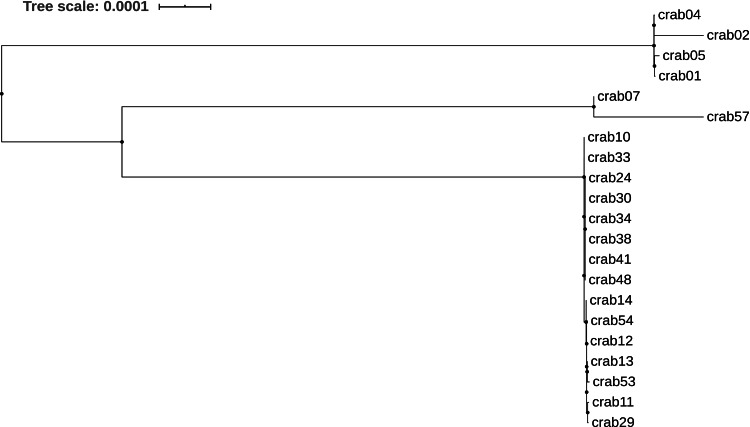
Maximum likelihood phylogenetic tree based on the SNPs in the core genomes of *Acinetobacter baumannii* clinical strains included in this study.

In conclusion, our study results indicate that, along with pursuing antimicrobial stewardship principles during COVID-19 pandemic, infection control activities targeted against the spread of antimicrobial resistance within and between hospitals should be revised and if necessary, remodulated according to the new organizational structures imposed by the pandemic.

## Supplementary material

For supplementary material accompanying this paper visit http://dx.doi.org/10.1017/ice.2021.144. click here to view supplementary material
