# Supplementary material for: Carbapenem-resistant bacteria in an intensive care unit during the coronavirus disease 2019 (COVID-19) pandemic: A multicenter before-and-after cross-sectional study
Source: Infect Control Hosp Epidemiol. 2021 Apr 16:1–6. doi: 10.1017/ice.2021.144 (PMC8365044; doi:10.1017/ice.2021.144)
Supplement: Supplementary file 1 [file S0899823X21001446sup001.docx]

| **Supplementary Table 1.** **Characteristics of ICUs before and during COVID-19 pandemic** | | | | |
| --- | --- | --- | --- | --- |
| **Hospital** | **ICU** | **Type** | **Bed number** | **Staff** |
| **Period 1: Jan-Apr 2019** | | | | |
| **PSO** | ICU1 | Medical and post-general surgery (mainly abdominal) | 22 | 40 intensivist specialists/residents  68 nurses  25 healthcare assistants |
|  | ICU-2 | Post-cardio-thoracic and vascular surgery | 21 | 43 intensivist specialists/residents  68 nurses  11 healthcare assistants |
| **MH** | ICU-3 | Post-traumatic resuscitation unit and HEMS | 11 | 21 intensivist specialists/residents  30 nurses  4 healthcare assistants |
|  | ICU-4 | Medical and post- surgery unit | 9 | 9 intensivist specialists/residents  25 nurses  4healthcare assistants |
| **BH** | ICU-5 | Neurology and neuro-surgery unit | 12 | 20 intensivist specialists/residents  35 nurses  5 healthcare assistants |
| **Period 2: Jan-Apr 2020** | | | | |
| **PSO** | ICU-1 | COVID Unit | 25 | 44 intensivist specialists/residents  91 nurses  20 healthcare assistants |
|  | ICU-1 bis | Small dedicated post-abdominal surgery (non-COVID) | 6 |  |
|  | ICU-2 | COVID Unit | 35 | 45 intensivist specialists/residents  90 nurses  25 healthcare assistants |
|  | ICU-2 bis | Small dedicated post-cardio-thoracic and vascular surgery (non-COVID) | 8 |  |
| **MH** | ICU-3 | Post-traumatic resuscitation unit and HEMS | 11 | 21 intensivist specialists/residents  25 nurses  4 healthcare assistants |
|  | ICU-4 | COVID Unit | 10 | 10 intensivist specialists/residents  20 nurses  4 healthcare assistants |
|  | ICU-4 bis | Medical and post- surgery unit-bis | 10 | 7 intensivist specialists/residents  20 nurses  3 healthcare assistants |
| **BH** | ICU-5 | COVID Unit | 26 | 25 intensivist specialists/residents  109 nurses  24 healthcare assistants |

Abbreviations: BH, Bellaria Hospital; HEMS, helicopter emergency medical service; ICU, Intensive Care Unit; MH, Maggiore Hospital; PSO, Policlinico Sant’Orsola;

**Supplementary Table 2. Phenotypic and genotypic characteristics of *Acinetobacter baumannii* strains included in this study**

|  |  |  |  |  | **MIC (µg/ml)** | | | | | |  | **Genetic determinants** | | | | | | | | | | |
| --- | --- | --- | --- | --- | --- | --- | --- | --- | --- | --- | --- | --- | --- | --- | --- | --- | --- | --- | --- | --- | --- | --- |
| **Isolate** | **Date of isolation** | **Hospital** | **Ward** | **Sample**  **Type** | **IPM** | **MEM** | **COL*** | **GEN** | **CIP** | **SXT** | **ST** | **Beta-lactams** | |  | **Aminoglycosides** | | |  | **Sulfonamide** | |  | **Phenicol** |
|  |  |  |  |  |  |  |  |  |  |  |  | bla*_OXA-23_* | bla*_TEM1-D_* |  | *armA* | *aph(3'')-Ib [aadA1]* | *aph(3'')-Ia* |  | *sul1* | *sul2* |  | *catB8* |
|  |  |  |  |  |  |  |  |  |  |  |  |  |  |  |  |  |  |  |  |  |  |  |
| CRAB01 | 05/04/2020 | MH | ICU-3 | Blood | 16 | ≥16 | 1 | ≥16 | ≥4 | ≥320 | 369 | X |  |  | X | X | X |  | X |  |  | X |
| CRAB02 | 23/04/2020 | BH | ICU-5 | BAL | ≥16 | ≥16 | 0.25 | ≥16 | ≥4 | 160 | 369 | X |  |  | X | X | X |  | X |  |  | X |
| CRAB04 | 07/04/2020 | BH | ICU-5 | BAL | ≥16 | ≥16 | 0.5 | ≥16 | ≥4 | 80 | 369 | X |  |  | X | X | X |  | X |  |  | X |
| CRAB05 | 07/04/2020 | MH | ICU-3 | BAL | ≥16 | ≥16 | 0.5 | ≥16 | ≥4 | 160 | 369 | X |  |  | X | X | X |  | X |  |  | X |
| CRAB07 | 15/04/2020 | MH | ICU-3 | BAL | ≥16 | ≥16 | 1 | ≥16 | ≥4 | ≥320 | 369 | X | X |  | X | X | X |  |  | X |  |  |
| CRAB10 | 03/04/2020 | PSO | ICU-1 | BAL | ≥16 | ≥16 | 0.5 | ≥16 | ≥4 | <2 | 195 | X | X |  | X | X | X |  |  |  |  |  |
| CRAB11 | 06/04/2020 | PSO | ICU-2 | BAL | ≥16 | ≥16 | 0.5 | ≥16 | ≥4 | ≥320 | 195 | X |  |  | X | X |  |  |  | X |  |  |
| CRAB12 | 15/04/2020 | PSO | ICU-2 | BAL | ≥16 | ≥16 | 2 | ≥16 | ≥4 | ≥320 | 195 | X | X |  | X | X | X |  |  | X |  |  |
| CRAB13 | 11/04/2020 | PSO | ICU-2 | BAL | ≥16 | ≥16 | 0.5 | ≥16 | ≥4 | 160 | 195 | X | X |  | X | X | X |  |  | X |  |  |
| CRAB14 | 11/04/2020 | PSO | ICU-2 | BAL | ≥16 | ≥16 | 1 | ≥16 | ≥4 | <2 | 195 | X | X |  | X | X | X |  |  | X |  |  |
| CRAB24 | 16/04/2020 | PSO | ICU-2 | Blood | ≥16 | ≥16 | 2 | ≥16 | ≥4 | ≥320 | 195 | X | X |  | X | X | X |  |  |  |  |  |
| CRAB29 | 17/04/2020 | PSO | ICU-2 | Blood | ≥16 | ≥16 | 1 | ≥16 | ≥4 | ≥320 | 195 | X |  |  | X | X |  |  |  | X |  |  |
| CRAB30 | 17/04/2020 | PSO | ICU-1 | Blood | ≥16 | ≥16 | 1 | ≥16 | ≥4 | <2 | 195 | X | X |  | X | X | X |  |  |  |  |  |
| CRAB33 | 21/04/2020 | PSO | ICU-2 | Blood | ≥16 | ≥16 | 1 | ≥16 | ≥4 | <2 | 195 | X | X |  | X | X | X |  |  |  |  |  |
| CRAB34 | 21/04/2020 | PSO | ICU-2 | BAL | ≥16 | ≥16 | 1 | ≥16 | ≥4 | <2 | 195 | X | X |  | X | X | X |  |  |  |  |  |
| CRAB38 | 27/04/2020 | PSO | ICU-1 | BAL | ≥16 | ≥16 | 1 | ≥16 | ≥4 | <2 | 195 | X | X |  | X | X | X |  |  |  |  |  |
| CRAB41 | 27/04/2020 | PSO | ICU-1 | BAL | ≥16 | ≥16 | 0.5 | ≥16 | ≥4 | <2 | 195 | X | X |  | X | X | X |  |  |  |  |  |
| CRAB48 | 07/04/2020 | PSO | ICU-1 | Blood | ≥16 | ≥16 | 1 | ≥16 | ≥4 | <2 | 195 | X | X |  | X | X | X |  |  |  |  |  |
| CRAB53 | 11/04/2020 | PSO | ICU-2 | Blood | ≥16 | ≥16 | 2 | ≥16 | ≥4 | ≥320 | 195 | X | X |  | X | X | X |  |  | X |  |  |
| CRAB54 | 14/04/2020 | PSO | ICU-2 | BAL | ≥16 | ≥16 | 0.5 | ≥16 | ≥4 | 160 | 195 | X | X |  | X | X | X |  |  | X |  |  |
| CRAB57 | 20/04/2020 | PSO | ICU-2 | Blood | 16 | ≥16 | 0.5 | ≥16 | ≥4 | ≥320 | 369 | X |  |  | X | X | X |  | X |  |  | X |

Abbreviations: BAL, Broncho Alveolar Lavage; BH, Bellaria Hospital; CIP, ciprofloxacin; GEN, gentamicin; ICU, Intensive Care Unit; IPM, imipenem; MH, Maggiore Hospital; MEM, meropenem; MIC, minimum inhibitory concentration; NA, not available; PSO, Policlinico Sant’Orsola; SXT, Trimethoprim–Sulfamethoxazole; ST, sequence type.

*Colistin MIC was evaluated by broth microdilution (BMD) method

**Supplementary Table 3. Characteristics of patients found to be colonized or infected with the CR-Ab strains assessed for clonal relationship.**

| **Patient ID** | **ICU** | **Patient provenance** | **Time from ICU admission to first CR-Ab isolation (days)** | **CR-Ab infection**  **(y/n)** | **Therapeutic management of COVID-19** | **Outcome** |
| --- | --- | --- | --- | --- | --- | --- |
| CRAB01 | ICU-3 | Surgical ward MH | 8 | N | Steroids, AZI, DRV/r | death |
| CRAB02 | ICU-5 | Medical ward BH | 36 | N | HCQ/CQ,DRV/r, LMWH,TOC,Steroids, AZI | Hospital discharge |
| CRAB04 | ICU-5 | Medical ward BH | 49 | Y  (Pneumonia) | HCQ/CQ, DRV/r, LMWH, TOC,Steroids, AZI | Hospital discharge |
| CRAB05 | ICU-3 | Surgical ward MH | 8 | N | Steroids, AZI, DRV/r | death |
| CRAB07 | ICU-3 | Medical ward MH | 12 | N | HCQ/CQ, DRV/r, LMWH, TOC, Steroids | Hospital discharge |
| CRAB10 | ICU-1 | ICU other Hospital (Piacenza) | 8 | Y  (Pneumonia) | HCQ/CQ,RDV,AZI, LMWH | Hospital discharge |
| CRAB11 | ICU-2 | Medical ward PSO | 12 | Y  (Pneumonia) | HCQ/CQ, LMWH, TOC, Steroids, AZI | death |
| CRAB12 | ICU-2 | ICU other Hospital (Piacenza) | 11 | N | HCQ/CQ, DRV/r, LMWH, TOC,Steroids | Hospital discharge |
| CRAB13 | ICU-2 | ICU other Hospital (Piacenza) | 23 | Y  (Pneumonia and BSI) | HCQ/CQ, DRV/r, LMWH, TOC,Steroids, AZI | death |
| CRAB14 | ICU-2 | Medical ward PSO | 17 | Y  (Pneumonia) | HCQ/CQ, DRV/r, LMWH, TOC, AZI | death |
| CRAB24 | ICU-2 | Medical ward PSO | 8 | N | HCQ/CQ, TOC, Steroids, AZI | death |
| CRAB29 | ICU-2 | ICU other Hospital (Piacenza) | 16 | N | HCQ/CQ, DRV/r, TOC, AZI | death |
| CRAB30 | ICU-1 | Medical ward PSO | 14 | N | HCQ/CQ, LMWH, TOC | Hospital discharge |
| CRAB33 | ICU-2 | Medical ward PSO | 26 | N | HCQ/CQ, DRV/r, TOC | Hospital discharge |
| CRAB34 | ICU-2 | ICU other Hospital (Piacenza) | 24 | Y  (Pneumonia) | LMWH, Steroids, AZI | Hospital discharge |
| CRAB38 | ICU-1 | Medical ward PSO | 25 | Y  (Pneumonia) | HCQ/CQ, TOC, Steroids, AZI | death |
| CRAB41 | ICU-1 | Medical ward other Hospital (Bentivoglio) | 21 | N | HCQ/CQ, LMWH, TOC | Hospital discharge |
| CRAB48 | ICU-1 | Medical ward PSO | 11 | N | LMWH, TOC | Hospital discharge |
| CRAB53 | ICU-2 | ICU other Hospital (Imola) | 20 | N | HCQ/CQ, LMWH | Hospital discharge |
| CRAB54 | ICU-2 | ICU other Hospital (Piacenza) | 23 | N | LMWH,TOC,Steroids | death |
| CRAB57 | ICU-2 | Medical ward PSO | 25 | N | HCQ/CQ, LMWH, TOC | Hospital discharge |

Abbreviations: AZI, azithromycin; BH, Bellaria Hospital; BSI, blood stream infection, CR-Ab, carbapenem resistant Acinetobacter baumannii; HCQ/CQ, hydroxycloroquine/chloroquine; ICU, Intensive Care Unit; LMWH, low-molecular-weight heparin; MH, Maggiore Hospital; PSO, Policlinico Sant’Orsola; RDV, remdesivir; TOC: tocilizumab;
